# Two Nigerian States (Plateau and Nasarawa) Have Eliminated Transmission of Human Onchocerciasis—A Report of Post-Ivermectin Mass Drug Administration Surveillance

**DOI:** 10.4269/ajtmh.22-0491

**Published:** 2022-11-30

**Authors:** Emmanuel S. Miri, Abel Eigege, Barminas Kahansim, Kenrick Nwodu, Yohana Sambo, Bulus Mancha, Solomon Adelamo, John Umaru, Jonathan Kadimbo, Jacob Danboyi, Hayward Mafuyai, Emeka Makata, Nse Akpan, Joel Akilah, Michael Igbe, Jenna Coalson, Lindsay Rakers, Emily Griswold, Thomas R. Unnasch, B. E. B. Nwoke, Gregory S. Noland, Frank O. Richards

**Affiliations:** ^1^ The Carter Center Nigeria, Jos, Nigeria;; ^2^ Plateau State Ministry of Health, Jos, Nigeria;; ^3^ Nasarawa State Ministry of Health, Lafia, Nigeria;; ^4^ University of Jos, Jos, Nigeria;; ^5^ Department of Public Health, Federal Ministry of Health, Abuja, Nigeria;; ^6^ The Carter Center, Atlanta, Georgia;; ^7^ University of South Florida, Tampa, Florida;; ^8^ Imo State University, Owerri, Nigeria

## Abstract

Transmission of *Onchocerca volvulus* (causing “river blindness”) was interrupted in two states of Nigeria (Plateau and Nasarawa) in 2017 in accordance with 2016 WHO guidelines. Ivermectin mass drug administration was halted in January 2018, and posttreatment surveillance activities were conducted over a 3-year period. Vector *Simulium damnosum* s.l. flies were collected during the 2019 (39 sites) and 2020 (42 sites) transmission seasons. Head pools were tested by polymerase chain reaction for the presence of third-stage *O. volvulus* larvae; 15,585 flies were all negative, demonstrating an infective rate of < 1/2,000 with 95% confidence. In 2021, the Nigerian Federal Ministry of Health declared the two-state area as having eliminated transmission. Plateau and Nasarawa states are the first of 30 endemic states in Nigeria to have met the WHO criteria for onchocerciasis elimination. Post-elimination surveillance will need to continue given the risk of reintroduction of transmission from neighboring states.

Human onchocerciasis (“river blindness”) is an infection caused by the filarial nematode parasite *Onchocerca volvulus*.^1^ Fertilized female *O. volvulu*s release microfilariae (mf) that migrate in the eye and subdermis, causing reactions that result in eye disease, a variety of skin lesions, and intense itching. The parasite is transmitted by certain species of *Simulium* black flies, with the most common vector being *S. damnosum* sensu lato (s.l.). In the vectors, ingested mf eventually develop into third stage larvae (L3) that migrate to the head of the flies. These can be infectious to humans when the flies take subsequent blood meals. There are no known environmental or epidemiologically important animal reservoirs of *O. volvulus*.

Mass drug administration (MDA) with ivermectin (Mectizan^®^, donated by Merck & Co., Inc., Lebanon, NJ) is the WHO-recommended strategy for the control or elimination of onchocerciasis.^2^ Ivermectin is a potent microfilaricide that also affects, in a limited way, the longevity and reproductive capabilities of the adult worms that normally live 8 to 14 years. Female worms are unable to release new mf into the skin for 3 to 6 months after ivermectin treatment. This means disease elimination strategies that use ivermectin MDA require repeated 6- to 12-month MDA cycles for many years. Disease transmission models estimate that after about 20 treatment rounds adult parasite populations collapse, thereby permanently interrupting transmission.^3^ At that point the MDA treatment program could be stopped, but well-planned posttreatment surveillance (PTS) is needed to detect recrudescence or reintroduction.

In 2017, Plateau and Nasarawa States were the first in Nigeria to meet the WHO guidelines for declaring the *interruption* of transmission of onchocerciasis and stopping ivermectin.^4^^,^^5^ This success was achieved by onchocerciasis programs that provided annual MDA from 1992 to 2017 with good coverage (a cumulative total of 27.2 million MDA treatments) over 25 to 26 years in the 12 hyper- and meso-endemic districts (local government areas [LGAs]). In addition, another 36.1 million cumulative MDA treatments over 7 to 12 years (2000–2012) of ivermectin (in combination with albendazole) for lymphatic filariasis was provided in the remaining 18 LGAs in the two states that were either nonendemic or hypoendemic for onchocerciasis.^4^^,^^6^^,^^7^ Here we provide results from the 3-year PTS period that will focus on the entomological evaluations required by WHO guidelines to declare transmission elimination.

The details of collection activities did not vary from the 2019 report by Richards et al.^4^ The survey protocol was approved by the state ministries of health of Plateau and Nasarawa and the Emory Institutional Review Boards, which considered it not to be human subject research, and standard monitoring and evaluation for a public health program. *S damnosum* s.l. black fly collections were conducted within 3 km of 39 to 42 villages (21 villages in Plateau in 2019 and 2020, 18 villages in Nasarawa in 2019, and 21 villages in Nasarawa in 2020) (Figure 1) during the peak blackfly breeding/biting seasons (mid-June through October) in 2019 (2 years after stopping MDA) and 2020 (3 years after stopping MDA). Thirty-nine village collection sites (93%) were the same sites examined in the original 2017 stop-MDA assessments: these included 33 villages required by the Nigeria National Onchocerciasis Elimination Committee (NOEC)^8^ and six villages used as sentinels for serial monitoring by the program (three in each state). The sentinel villages had a mean 1991 baseline skin snip mf prevalence in adult males of 64% (range 51–93%).^4^ In 2020, three special PTS villages (labeled in Figure 1) were newly selected because two were situated within 3 km of the border with Benue State (classified in 2018 by the NOEC as “onchocerciasis transmission ongoing”) and one near Taraba State (NOEC classified as “on track for elimination”). The other four territories and states bordering with Plateau and Nasarawa are Federal Capital Territory (“on track for elimination”), Bauchi state (“transmission suspected to be interrupted”), and Kaduna state (“transmission interrupted”).

**Figure 1.**
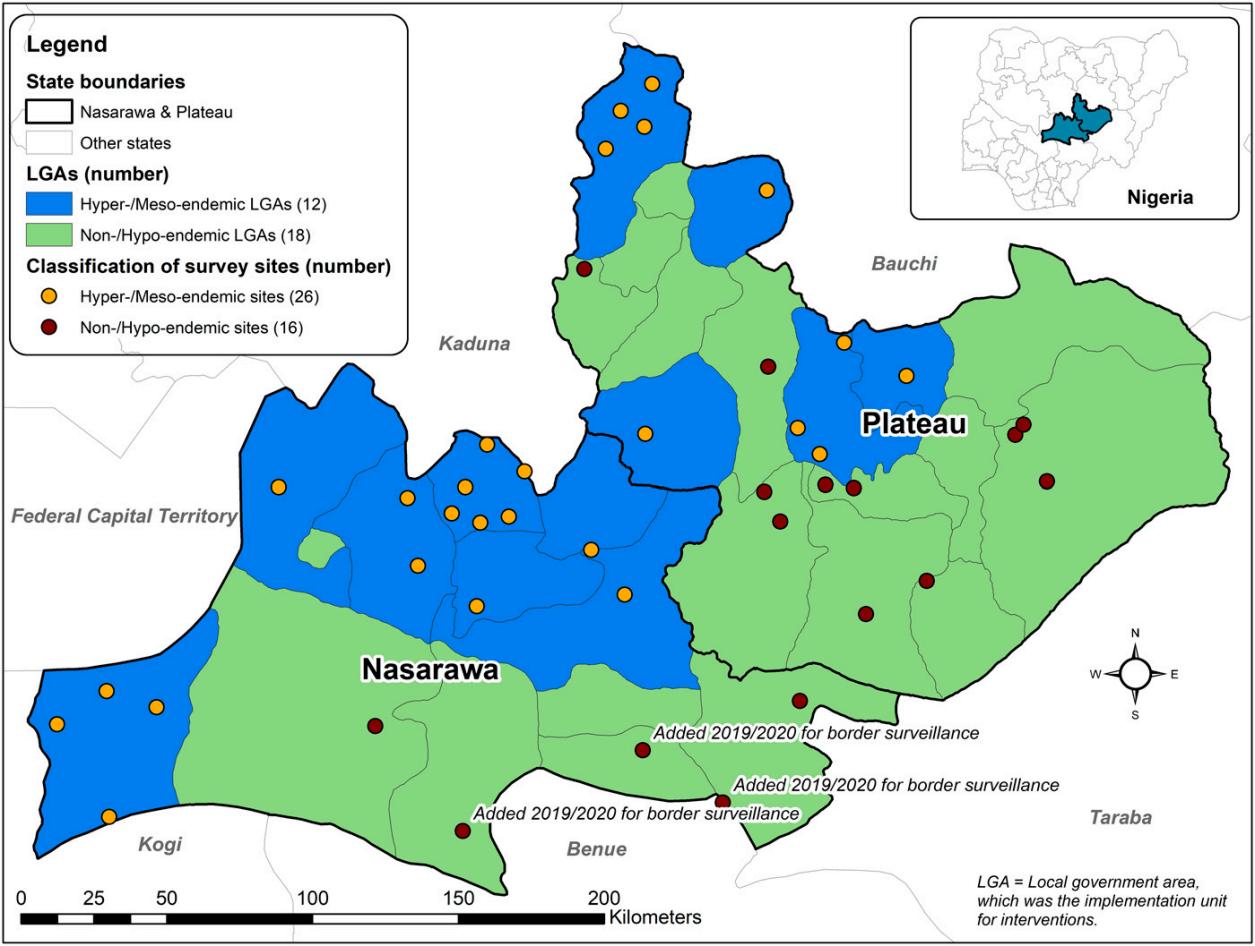
Dots represent 42 villages in Plateau and Nasarawa states of central Nigeria where entomological surveillance for onchocerciasis was conducted in 2019 and 2020. Names of the surrounding states are shown. Three new survey sites in Nasarawa, located near the Benue and Taraba state borders, are labeled.

Two human landing capture (HLC) sites were established in known or potential local *Simulium* vector-breeding points within 3 km of each selected village. Each site had four capture days per month, consisting of hourly collections (50 minutes of catches and 10 minutes of rest) between 7:00 am and 5:50 pm. Fly collectors exposed their lower limbs and sat quietly with bijou bottles (“tubes”) awaiting the vector flies. Flies were not allowed to bite. Daily catches were pooled at the site, preserved in isopropanol, and labeled with the site and date. All collectors were offered a 150 μg/kg dose of ivermectin at the end of the seasonal catching period.

Two Esperanza window traps (EWT)^9^^,^^10^ were placed within 3 km of known or potential local breeding sites. Carbon dioxide was generated from a bakers’ yeast and sugar solution. Dirty clothes from HLC collectors were hung at the top of the trap to serve as human scent lure. Vector flies stuck to the surface of the trap by Tangle-Trap^®^ adhesive. These were removed with solvent, combined in a tube, and preserved in isopropanol. Each collection tube was labeled with the site and date of fly removal from the trap.

The tubes were sent to the Carter Center laboratory in Jos, Plateau State, where the preserved flies were confirmed under a dissecting microscope to be *S. damnosum* s.l., then pooled in groups of up to 100 by village site. The heads were separated from the bodies and extracted DNA from head pools was tested for the O-150 repeat in polymerase chain reaction (PCR) analysis.^11^ The PCR products were detected by the OVS2fl probe and read using ELISA as previously described.^12^ Criteria for successful elimination of onchocerciasis transmission was a rate of infective black flies having a 95% upper confidence limit (UCL) of < 1/2,000.^5^ The 95% UCL in the O-150 black fly PCR analysis was calculated using PoolScreen 2.1 (Available at: https://www.soph.uab.edu/faculty/bst/charles_katholi).^13^

A total of 15,585 *S. damnosum* s.l. were captured and tested during the 2019 and 2020 transmission seasons. In Nasarawa State, 4,912 flies were captured in 2019 (1,772 [36%] by HLC and 3,140 [64%] by EWT) and 3,305 flies in 2020 (1551 [47%] by HLC and 1,754 [53%] by EWT). The combined total of 8,217 flies were tested in 93 pools that were all PCR negative (Table 1), yielding a 95% UCL of 0.48/2,000. In Plateau State, as observed in our previous report,^4^ HLC performance was considerably inferior to EWT: 3,517 flies were captured in 2019 (489 [14%] by HLC and 3,028 [86%] by EWT) and 3,851 flies were captured in 2020 (161 [4%] by HLC and 3,690 [96%] by EWT). A total of 7,368 flies were tested in 89 pools that were all PCR negative (Table 2), yielding a 95% UCL of 0.52/2,000. The states therefore individually met the 2016 WHO criteria for elimination of transmission of onchocerciasis requiring PTS entomology infectivity rates of < 1/2,000 (0.05%) with 95% confidence in a sample of at least 6,000 vectors.

**Table 1:** Nasarawa state: 2019 and 2020 transmission season results from O150 PCR analysis for *Onchocerca volvulus* in *Simulium damnosum* s.l. heads, collected from 18 sites in 2019 and 21 sites in 2020

Local government areas	Village	No. of black flies collected 2019*	No. of black flies collected 2020^†^	No. of Blackflies analyzed	No. of PCR pools Positive
Akwanga	Alushi	252	52	304	0
Akwanga	Anguwan Zaria	748	226	974	0
Akwanga	Anguwan Habu (SV)	764	280	1,044	0
Akwanga	Bayan Dutse (SV)	894	932	1,826	0
Akwanga	Gbuja	0	0	0	0
Awe	Wuse	0	16	16	0
Awe	Jangargari (NBV)	ND	0	0	0
Doma	Jirah (NBV)	ND	1	1	0
Karu	Jankanwa	5	10	15	0
Kokona	Gurku	260	40	300	0
Kokona	Nindama	1,733	303	2,036	0
Keana	Uyuwe (NBV)	ND	0	0	0
Nassarawa Eggon	Ezzen Sarki	62	210	272	0
Obi	Adudu	64	72	136	0
Toto	Akewa	115	93	208	0
Toto	Nyanji (SV)	15	1,070	1,085	0
Toto	Kuru	0	0	0	0
Toto	Umaisha	0	0	0	0
Toto	Moanya	0	0	0	0
Lafia	Arikiya	0	0	0	0
Lafia	Ugah	0	0	0	0
Total	–	4,912	3,305	8,217	0

NBV = new border state village in 2020; ND = no data; PCR = polymerase chain reaction; SV = sentinel village in 2020.

* Two years after stopping mass drug administration (MDA).

^†^ Three years after stopping MDA.

**Table 2:** Plateau state: 2019 and 2020 results from O150 PCR analysis for *Onchocerca volvulus* of *Simulium damnosum* s.l. heads, collected from 21 sites

Local government areas	Village	No. of blackflies collected 2019*	No. of blackflies collected 2020^†^	No. of Blackflies analyzed	No. of pools positive
Bassa	Mafara (SV)	0	3	3	0
Bassa	Lemoro (SV)	2,776	3,165	5,941	0
Bassa	Amokatako	3	2	5	0
Bassa	Majaja	0	13	13	0
Bokkos	Daffo	0	0	0	0
Jos-East	Godong (SV)	450	132	582	0
Kanke	Jinglai	0	6	6	0
Lantang South	Mabudi	80	270	350	0
Mangu	Fwangko	5	0	5	0
Mikang	Piapung	0	26	26	0
Mikang	Lifidi	16	0	16	0
Pankshin	Gung	7	0	7	0
Pankshin	Jing	0	0	0	0
Pankshin	Jivir	0	0	0	0
Quan'pan	Kwalla	4	0	4	0
Quan'pan	Bong	0	0	0	0
Riyom	Bum	25	7	32	0
Shendam	Shimankar	0	0	0	0
Wase	Sabongida mavo	0	0	0	0
Wase	Lamba	129	227	356	0
Wase	Gumshar	22	0	22	0
Total	–	3,517	3,851	7,368	0

PCR = polymerase chain reaction; SV = sentinel village in 2020.

* Two years after stopping mass drug administration (MDA).

^†^ Three years after stopping MDA.

In addition to entomological surveillance, other PTS activities included 1) a health communication campaign of more than 1,500 radio jingle slots and distribution of more than 10,000 health education posters (these had different messages for LGAs situated on a border compared with internal LGAs); 2) providing 21,994 ivermectin treatments to those having signs and/or symptoms of onchocerciasis in 300 villages located near state borders and in internally displaced person camps; and 3) cross-border coordination meetings between health facility staff, focused on Benue State, Taraba State, and Federal Capital Territory. As noted earlier, the NOEC in 2018 had declared transmission of onchocerciasis had been interrupted in Kaduna State and suspected it to be so in Bauchi State.

The NOEC reviewed these activities and the entomological surveillance results at its 12th meeting in May 2021 and recommended to the FMOH that transmission in these two states be declared eliminated.^14^ The FMOH accepted the recommendation soon thereafter and declared Plateau and Nasarawa States as the first to eliminate onchocerciasis transmission in Nigeria.

The approach to PTS in these two states could be considered by other endemic states in Nigeria as a template for their own post-MDA activities. However, PTS will need to be adapted to the context and realities of each state. For example, a state on an international border would need to take a very different approach related to importation of infection to that used in Plateau and Nasarawa.

Consistent with WHO guidelines, NOEC/FMOH recommended post-elimination surveillance (PES) due to the risk of reintroduction of the parasite by either infected humans or infected vectors migrating from other endemic states. PES would include the following: 1) continued vector surveillance and PCR testing every 3 years, including from the establishment of new sites in additional border villages; 2) ongoing health education campaigns with refined and targeted messages as well as new engagement of former community-directed drug distributors in the PES process to detect any new or unexpected concerns; 3) tracking and treating immigrants and internally displaced persons; and 4) expanded cross-border meetings with health authorities in all neighboring states. Sustained financial support from all partners for PES activities is important to maintain until (and probably beyond) the achievement of countrywide WHO verification of onchocerciasis transmission elimination from Nigeria.
